# Sinonasal Schwannoma with New Bone Formation Expressing Bone Morphogenic Protein

**DOI:** 10.1155/2010/154948

**Published:** 2010-12-19

**Authors:** Satoru Kodama, Tomoyo Okamoto, Masashi Suzuki

**Affiliations:** Department of Otolaryngology, Faculty of Medicine, Oita University, 1-1 Idaigaoka, Hazama-machi, Yufu, Oita 879-5593, Japan

## Abstract

Schwannoma is a benign tumor that arises from the sheath of myelinated nerve fibers and may occur in any part of the body. Osteogenesis in schwannoma is extremely rare and, to date, new bone formation in sinonasal schwannoma has not yet been reported. Here, we describe the first reported case of sinonasal schwannoma with new bone formation. The tumor was successfully treated by endoscopic sinus surgery, and the patient showed no evidence of recurrence 24 months postoperatively. Immunohistochemically, the tumor expressed bone morphogenic protein 4, indicating a possible role of this protein in the new bone formation in schwannomas.

## 1. Introduction

Schwannoma, also known as neurilemoma, is a benign tumor that arises from the sheath of myelinated nerve fibers and may occur in any part of the body. Schwannomas most commonly occurs in the head and neck region (approximately 25%–45% of cases) but rarely in the nose and sinuses [1–3]. Osteogenesis in schwannoma is extremely rare, and only a few cases of schwannoma with osteogenesis in the internal auditory canal, lacrimal gland, spine, or other soft tissues have been reported [4–9]. New bone formation in nasal polyps has been demonstrated [10, 11], and bone formation in sinonasal inverted papilloma has been reported recently [12, 13]; however, to date, new bone formation in sinonasal schwannoma has not been reported. Here, we describe the first reported case of sinonasal schwannoma with new bone formation. The tumor was successfully treated by endoscopic sinus surgery (ESS).

## 2. Case Report

**Figure 1:**
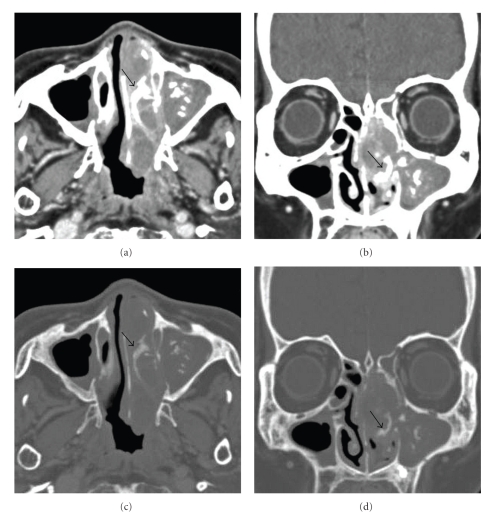
Horizontal (a) and axial (b) enhanced computed tomography (CT) showed an inhomogeneously enhancing mass that filled the left nasal cavity. A low-density lesion with metal-dense spots in the left maxillary sinus, indicating mycetoma, was also observed. Bone window images ((c), (d)) also showed a new bone formation in the tumor. Black arrows indicate a new bone formation.

**Figure 2:**
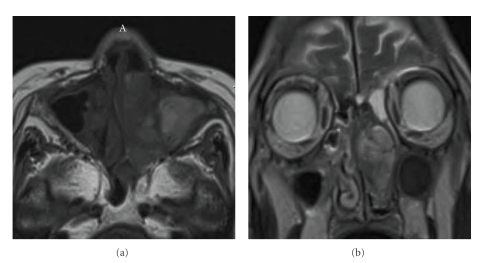
Horizontal (a) and axial (b) magnetic resonance imaging (MRI) showed a nasal tumor in the left nasal cavity with a hypointense signal on T1-weighted images, and a moderately hyperintense signals on T2-weighted images. This signal was different from that of the left maxillary sinus lesions.

**Figure 3:**
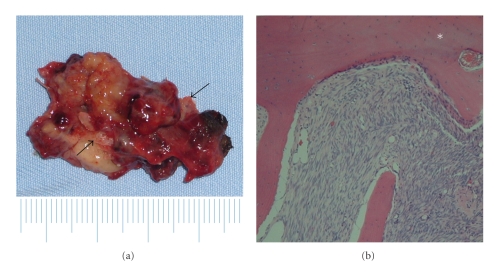
Macroscopic (a) and microscopic (x100 magnification) (b) features of the excised tumor. The spindle cells are arranged in a patternless fashion, and new bone formation consisting of randomly organized trabeculae lined by osteoblasts was observed in the tumor. black arrows and *asterisk: bone tissue.

An 81-year-old woman presented with a left nasal tumor that caused left nasal obstruction. Nasal endoscopy revealed a pinkish smooth mass that filled the left nasal cavity. Enhanced computed tomography (CT) showed an inhomogeneously enhancing mass that filled the left nasal cavity. The tumor grew expansively and showed marked osteogenesis (Figure 1). A low-density lesion with metal-dense spots in the left maxillary sinus, indicating mycetoma, was also observed. Magnetic resonance imaging (MRI) showed a nasal tumor in the left nasal cavity with a hypointense signal on T1-weighted images, and a moderately hyperintense signals on T2-weighted images (Figure 2). The signal of the nasal tumor was distinctly different from that of the left maxillary sinus lesions. Biopsy of the tumor was performed, and preoperative pathologic examination revealed schwannoma. No neurologic defects were observed preoperatively. ESS, which was selected on the basis of the preoperative radiologic findings, was performed under general anesthesia. Broad attachment of the tumor was observed in the superior nasal meatus and middle turbinate. Harmonic Scalpel, an ultrasonic dissector coagulator, and a suction curette were used to excise the mucosa with tumor involvement, along with a margin of macroscopically normal mucosa. Many olfactory fibers were identified and resected with the mucosa. The anterior ethmoid nerve was also identified and resected. Newly generated bone tissue was surrounded with the tumor. The tumor was thus successfully removed in one piece with the surgical margin free of disease (Figure 3(a)). Moreover, a fungus ball was completely cleared from the left maxillary sinus. The operation was performed for 1 hour, and the intraoperative blood loss was 20 ml. 

**Figure 4:**
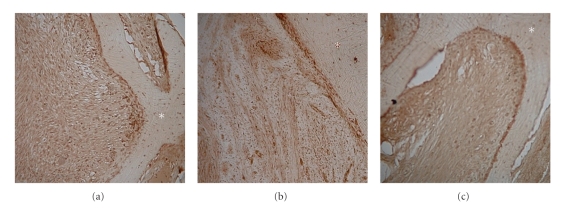
Immunohistochemistry for bone morphogenic protein (BMP; x100 magnification). (a) BMP-2, (b) BMP-4, and (c) BMP-7. The tumor cells stained positive for BMP-4 and negative for BMP-2 and BMP-7. *asterisk: bone tissue.

**Figure 5:**
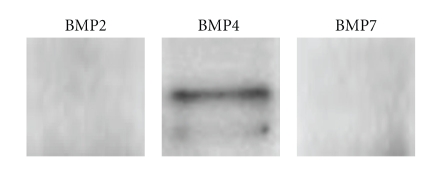
Western blot analysis for BMP. The tumor tissue expressed BMP-4 but not BMP-2 and -7.

 Histopathologically, a patternless proliferation of spindle cells and new bone formation consisting of randomly organized trabeculae lined by osteoblasts were observed in the tumor (Figure 3(b)). Immunohistochemically, the tumor cells stained strongly positive for S-100 proteins and negative for smooth muscle actin, desmin, and Ki-67. Thus, a definitive diagnosis of schwannoma with new bone formation was established. To further investigate the osteogenesis in the tumor, the expression of bone morphogenic proteins (BMPs) was examined. Rabbit polyclonal antibodies (Abs) against human BMP-2, BMP-4 (LifeSpan BioSciences, Seattle, WA), and BMP-7 (AVIVA Systems Biology, San Diego, CA) were used for the detection of BMP expression in the tumor. Four-micrometer-thick paraffin sections were prepared for light microscopic examination. After incubation with Abs against BMPs, sections were incubated with biotinylated antirabbit IgG (Vector Laboratories, Burlingame, CA). After rinsing, sections were incubated with ABC reagent (Vector Laboratories) and developed in 0.05% 3,3′-diaminobenzidine-0.01% H2O2 substrate medium in 0.1 M phosphate buffer. Interestingly, the tumor cells stained positive for BMP-4 and negative for BMP-2 and BMP-7 (Figure 4). Western blot analysis was also performed to confirm the expression of BMPs in the tumor. The tumor tissue lysates were subjected to SDS-PAGE. After electrophoresis, proteins were transferred onto a PVDF membrane (Millipore, Billerica, MA, USA). The blots were incubated with Abs against BMP-2, -4, and -7, same as immunohistochemistry. After washing, membranes were incubated with HRP-conjugated goat anti-rabbit IgG secondary Ab (Santa Cruz Biotechnology, Inc.). Protein bands were visualized with ECL substrate (GE Medical). The result showed the expression of BMP-4, but not BMP-2 and -7, in the similar manner to the result of immunohistochemistry (Figure 5). 

 Histopathologic examination also showed aspergillosis in the left maxillary sinus. The postoperative course was uneventful with no complications or neurologic defects. There was no recurrence at the 2-year followup examination.

## 3. Discussion

 Schwannoma commonly occurs in the head and neck region but rarely in the nasal cavity and sinuses [1–3]. In addition, new bone formation accompanying schwannoma is extremely rare, with only a few cases of schwannoma with calcification in the internal auditory canal, lacrimal gland, spine, and other softtissues having been reported [4–9]. To date, sinonasal schwannoma with new bone formation has not been reported. Here, we present the first case of sinonasal schwannoma with new bone formation and its clinical management.

 There have been three reported cases of bone formation in nasal polyps [10, 11]. In addition, four cases of the association of inverted papillomas and new bone formation have been reported recently [11, 12]. However, the mechanism of osteogenesis in these inflammatory and tumor tissues remains unclear. Calcifications may be classified as entrapped bone structures or as primary tumoral calcifications. Essentially, entrapped bone is the bone fragment, which is enclosed within the tumor and erodes due to pressure atrophy whereas primary tumorous calcification is calcification created by the tumor itself; both may appear as calcifications on CT [13]. The histopathologic examination revealed no entrapped bone or primary tumor calcification in the current bone structure. The examination showed new bone formation consisting of randomly organized trabeculae lined by osteoblasts. These randomly organized trabeculae were divided with prominent capillaries and mesenchymal cells. In general, however, the entrapped bone should be in a lamellar structure, but in our case, the bone trabeculae were woven and represented active osteoblast production of newly formed bone. In addition, intraoperative findings showed that the newly generated bone in the tumor was independent of normal anatomical bone structure. Thus, the current case was diagnosed as schwannoma with new bone formation.

 Clinically, schwannoma with accompanying new bone formation might appear to behave no differently from common schwannoma, and its features might be similar. The treatment of choice for sinonasal schwannoma is surgery. To date, less than 100 cases of sinonasal schwannoma have been reported [1–3], and different approaches, including lateral rhinotomy with external ethmoidectomy, the Caldwell-Luc approach, midfacial degloving, or ESS, have been employed in relation to tumor extension [3, 14]. ESS is now widely accepted and commonly performed in cases requiring nose or paranasal sinus surgery. ESS provides excellent magnification, illumination, and angled visualization, thereby allowing the surgeon to isolate the base of the tumor and accurately define the extent of disease. Long-term followup of our patient will reveal the optimal clinical management of sinonasal schwannoma with new bone formation.

 Another important finding in this paper is the demonstration of BMP expression in the tumor. This is the first paper showing BMP expression in schwannomas. BMPs are multifunctional growth factors belonging to transforming growth factor beta superfamily. It has been demonstrated that BMPs had been involved in the regulation of cell proliferation, survival, differentiation, and apoptosis [15]. However, their hallmark ability is that play a pivotal role in inducing bone, cartilage, ligament, and tendon formation at both heterotopic and orthotopic sites. Extensive studies demonstrate that BMPs are important factors regulating chondrogenesis and skeletogenesis during normal embryonic development [16]. The BMPs with greatest osteogenic capacity are BMP-2, -4, -5, -6, -7, and -9. BMP-2 acts as a disulfide-linked homodimer and induces bone and cartilage formation. It is candidate as a retinoid mediator and plays a key role in osteoblast differentiation. BMP-4 regulates the formation of teeth, limbs, and bone from mesoderm and also plays a role in fracture repair. BMP-7 plays a key role in osteoblast differentiation [15]. Expression of BMPs in tumor tissues has also been reported [17]. BMP 2/4 was localized predominantly to the cytoplasm of malignant cells with primitive mesenchymal features; no or little BMP is detected in the more differentiated elements of bone and softtissue sarcomas [17]. Different levels of BMP 2/4 expressions in bone and soft tissue sarcomas have been considered to be associated with the stage of mesenchymal differentiation. In the current case, the schwannoma cells expressed BMP-4 inhomogenously. Although the detail of role of BMP remains unclear in the pathogenesis in the current tumor, this indicates possible role of BMP-4 for new bone formation in schwannomas.

 In conclusion, we have described the first reported case of sinonasal schwannoma with new bone formation. ESS could be successfully used to achieve complete removal of the tumor. BMP-4 might be associated with new bone formation in the tumor.
